# Mucosal bacterial immunotherapy with MV130 highly reduces the need of tonsillectomy in adults with recurrent tonsillitis

**DOI:** 10.1080/21645515.2019.1581537

**Published:** 2019-04-17

**Authors:** Luis-Amando García González, Federico Arrutia Díez

**Affiliations:** a ENT Department, Hospital Valle del Nalón, Asturias, Spain; b ENT Department, Hospital de Cabueñes, Asturias, Spain

**Keywords:** Recurrent tonsillitis, tonsillectomy, infectious disease, oral mucosal immunotherapy

## Abstract

Recurrent tonsillitis in adults is a common ENT disease. The current standard treatment is tonsillectomy. However, continuous prophylaxis with antibiotics has been prescribed in order to avoid tonsillectomy. The objective was to evaluate if the bacterial immunotherapy (Bactek MV130) together with the prophylactic antibiotic therapy can produce clinical improvement and to avoid the tonsillectomy.

**Material and methods:** The medical records of 88 patients with recurrent tonsillitis were reviewed. Sixty-six were treated during 3 months with a course of antibiotics and 22 received, in addition to the antibiotics, immunotherapy with Bactek MV130 during this Globally, 53 (60%) patients had clinical improvement and 35 were tonsillectomized. In the The group of patients who received only antibiotic, 35 (53%) avoided tonsillectomy and 31 (47%) did not. In the group that, in addition to antibiotics, were treated with Bactek MV130, 18 patients (82%) experi- enced clinical improvement avoiding tonsillectomy and 4 (18%) didn’t improve and the tonsils were surgically removed. The difference between both groups was significant (P = 0.023).he results obtained in this evaluation support this combined treatment as an effective strategy to reduce the need of tonsillectomy.

## Introduction

Recurrent tonsillitis is a common otorhinolaryngological disease, with a prevalence rate of 11 to 12.3%. Recurrent tonsillitis was defined as four or more episodes of tonsillitis per year with two of the episodes confirmed to be group A streptococcal infection.^1^ The diagnosis is mainly based on the patient’s clinical history, usually associated with sore and chronic scratchy throat and systemic symptoms such as fever, malaise, painful tonsillar microabscesses and cervical lymphadenopathy with or without peritonsillar erythema. It is a clinical issue that persists for more than 3 consecutive months despite medical treatment.^2^

Currently, the standard therapeutic approach is tonsillectomy.^3^ The goal of this surgical intervention is the disappearance of chronic inflammatory processes, a fact which has a positive influence on the quality of life of patients and minimizes health costs. This intervention produces a significant improvement in quality of life, decreases health-care utilization and diminishes the economic burden of chronic tonsillitis in the adult patient.^4^Although this intervention is common in the field of Ear, Nose and Throat (ENT) surgical interventions and it is usually considered as minor, the evolution of post-tonsillectomy in adult patients is more torpid than in childhood. Complications and risks of tonsillectomy include pain, nausea, vomiting, hemorrhage, dehydration, admission for postoperative oxygen desaturations.^5^ Rare complications include intraoperative vascular injury, subcutaneous emphysema, mediastinitis, Eagle’s syndrome, atlantoaxial subluxation, cervical osteomyelitis, and taste disorders.^6^ Mortality rates for the operation range from 1 in 10,000 to 1 in 35,000^7^, usually as a result of postoperative bleeding.

Immunotherapy with agents of bacterial origin have been used as complementary treatment in patients with recurrent infections due to their actions on the immune system: boosting innate immunity and increasing immune responses.^8^ Studies have shown that the oral administration of preparations of bacterial origin ameliorates recurrent infections in adults and children by reducing the number, duration and severity of these episodes.^9^ Bacterial preparations administered through the sublingual route have demonstrated to be safe and effective, inducing a strong and long-lasting stimulation of mucosal and systemic non-specific and antigen-specific cell-mediated and humoral immunity.^10^

Bactek consisted of a suspension of 300 Formazin Turbidity Units (FTU) of inactivated whole bacteria/mL (equivalent to 10^9^ bacteria/mL), containing a mixture of selected strains of the bacteria frequently present in infected tonsils and in the oropharyngeal mucosa^11^ (Staphylococcus aureus, Staphylococcus epidermidis, Streptococcus pneumoniae, Haemophilus influenza, Streptococcus pyogenes).

This study is our clinical experience of patients with recurrent tonsillitis who definitely were candidates for tonsillectomy and were treated with intramuscular 1,200,000 International Units (IU) of penicillin or azithromycin (only in patients allergic to penicillin) before surgery with or without bacterial immunotherapy with Bactek and to evaluate the clinical benefit of these preparations in order to avoid the surgery (tonsillectomy).

## Results and discussion

### Patients

The number of medical records screened, discarded and accepted for the evaluation is shown in Figure 1. There were no differences (*P* > 0.05) between both groups of patients regarding age, the median of age (with Interquartile range) was 25 years (IQ range 20–31), gender and the number of episodes of tonsillitis in the previous 12 months before the initiation of treatment with the course of antibiotic with or without Bactek® (Table 1).

**Table 1.** Demographic data of patients.

	No Bactek	Bactek	*P*
n	66	22	
Age (years)	24 (20,0-30,0)	29 (22,8-35,5)	0,0799#
Episodes	4(4-5)	5 (4-6)	0,0610#
Male/Female	18/48	6/16	>0,9999##

# Mann-Whitney’s test

## Fisher’s exact test

**Figure 1.**
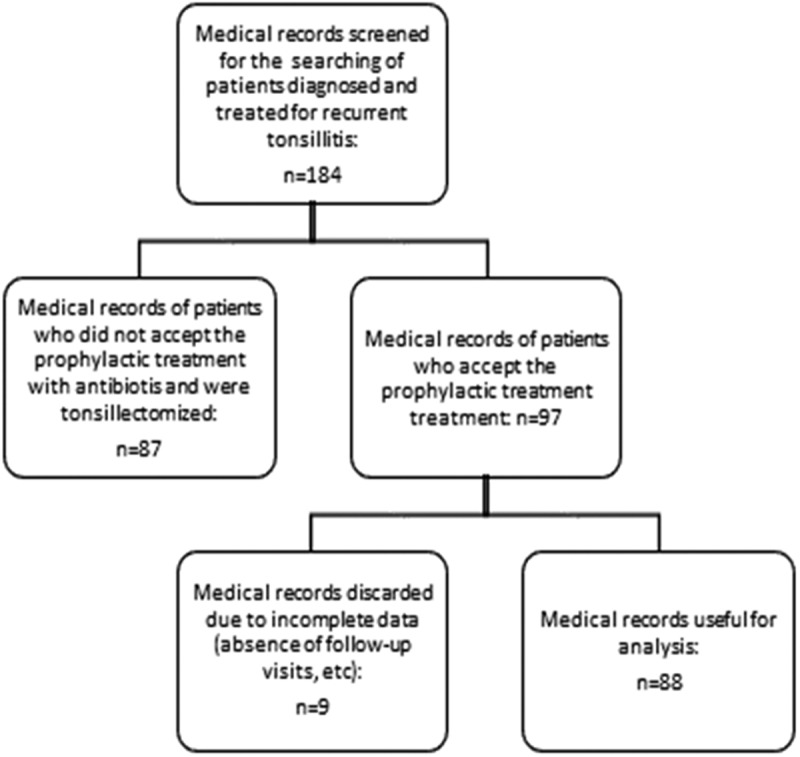
Flow chart of the study.

### Clinical benefit

Globally, 53 (60%) had improvement and 35 (40%) did not and were tonsillectomized. In the group of patients receiving prophylaxis solely with antibiotic, 35 (53%) had a good evolution and could avoid tonsillectomy, whereas 31 (47%) had a minimum of 2 new episodes of tonsillitis and required tonsillectomy. In the group-receiving antibiotic and Bactek®, 18 patients (82%) showed clinical improvement, avoiding tonsillectomy and 4 (18%) did not improve and were tonsillectomized. The difference between both groups was significant (*P* = 0.0230, Fisher’s exact test) with a power of 70% and an odds ratio of 3.99 (1.28, 12.42). Results are summarized in Table 2. The macroscopic examination of the tonsils of patients that avoided tonsillectomy revealed that were less hyperemic, with fewer crypts and had lower caseum retention.

**Table 2.** Patients that were tonsillectomized or not after the course of treatment with antibiotics with or without Bactek.

	No Bactek	Bactek	*P* (Fisher’s exact test)
Yes	31 (47%)	4 (18%)	0,0230**
No	35 (53%)	18 (82%)	

### Safety and compliance

None of the patients reported any side effect regarding Bactek®, either local at the site of administration or systemic. All patients completed the treatment.

In this manuscript we describe our experience in the clinical evolution of 88 patients, diagnosed of recurrent tonsillitis with chronic inflammation. BThe majority of the patients (82%) who received immunotherapy with Bactek experienced clinical improvement, whereas in the other group the percentage of patients was 51%.

To the best of our knowledge, this is the first communication of the use of immunotherapy with a bacterial preparation to successfully treat recurrent tonsillitis in adults avoiding surgery. Aznabaeva et al^12^ reported that the local therapy with bacterial lysates produced an activation of innate immunity and a normalization in the production of antibodies. However, they didn’t report clinical effectiveness regarding the need of tonsillectomy. In a retrospective study in children with recurrent tonsillitis, Bitar^13^, using an oral polybacterial lysate, reported a complete clinical effectiveness in 51.2% of the patients.

The use of immunotherapy with bacterial preparations as a prevention of recurrent infections of the upper respiratory tract, including ear, nose and throat, is well documented.^9^ The majority of the products used in these studies contain bacterial lysates that are administered through the oral route (swallowed).

The route of administration (sublingual), the different bacterial formulation and/or strains and the form in which the bacteria are harvested, manufactured and delivered (bodies instead of lysates) might explain this difference in the clinical benefit.^14,15^

It is emphasized that purified components from bacteria or bacterial lysates, selectively activate specific Toll Like Receptors (TLR), leading to shared and unique responses in innate immune cells,^15^ whereas whole bacteria contain multiple agonists for multiple TLR, eliciting a potent and robust response.^16^

The sublingual mucosa is a good inductive site for generating broad spectrum of mucosal and systemic immune responses, with a high degree of efficacy and persistence of the immune response^17^ in the respiratory and genitourinary tracts.^18,19^ Furthermore, it has been demonstrated that the sublingual administration of immunogens^18^ and whole bacteria^20^ activates dendritic cells and induces systemic dose-dependent immune responses, generating Th1, Th17 and IL-10 responses.^20^ The preparation used in this study is delivered sublingually, by lifting the tongue and spray it on its down side. Thus, both lingua and sublingual mucosa surfaces are treated in a rather high area, in which a high number of antigen presenting cells (dendritic cells) continuously sampling the lumen are found.^21^

An important issue in this study is the reduction in the expenses related to tonsillectomy. The direct cost of tonsillectomy is approximately 1,600 €, the use of hospital facilities is 685 € daily and 49 € each follow-up visit.^22,23^ Other indirect economic costs are those related to the loss of working days and school absenteeism.

The use of this immunotherapy approach is in line with the advice of the health organizations (WHO, EMA, FDA, …) for seeking new treatment alternatives against bacterial diseases, given the rise and spread of antibiotic resistant bacteria jeopardy.

We acknowledge that the study has major limitations such the lack of immune status and co-morbid conditions. However, Because this study is a compilation of our experience in a small group of patients, it does not provide deeper outcomes as could be obtained in an explanatory prospective and randomized double-blind placebo-controlled trial. However, one cannot dismiss that our experience provides valuable information of the effectiveness of this treatment under “real life” conditions and identifies the feasibility of a prospective study in adult patients with recurrent tonsillitis, who are candidates for tonsillectomy, according to the current state of the art.

Considering the clinical impact of this pathology, together with the increasing resistance to antibiotics, the economic costs and the non-negligible morbidity of adult tonsillectomy, the results obtained in this study supports to conduct more studies in order to evaluate if this treatment could be an effective strategy to reduce the need of tonsillectomy, but we are aware that a larger sample would allow greater statistical strength.

## Material and methods

### Patient population

It was a retrospective pilot study based on medical records review. These were from of our otolaryngological office and all were related to patients diagnosed of recurrent tonsillitis in the last 5 years and who were, definitely, candidates for tonsillectomy. Finally, the medical records of 88 patients (24 men and 64 women), treated before surgery with a prophylactic course of antibiotic with or without immunotherapy with bacterial preparation, were analyzed. All patients had recurrent tonsillitis with chronic inflammation, suffering a minimum of 4 acute episodes in the last 12 months. In all cases, the exploration revealed that tonsils were hyperemic and with the presence of crypts, caseum retention and congestive soft palate. The palpation demonstrated fibrous capsule tonsils strongly adhered to pharyngeal muscle. All these patients were previously treated with antibiotics and anti-inflammatory drugs without reaching control of the recurrent infections, considering it as a treatment failure. No patients had previous episodes of peritonsillar abscess o flemonous tonsillitis. In the 88 treated patients, 23 cases had torpid tonsillitis episodes that required hospitable entry.

### Treatment

Following the recommendations of six Spanish Scientific Societies outlined in a Consensus Document^24^, 66 patients were treated with an antibiotic course: 61 received 4 monthly intramuscular injections of benzathine penicillin (1,200,000 units/injection) and 5, who were allergic to penicillin, received Azithromycin (500 mg during 3 days, every 2 weeks during 1 month.

The rest of patients^22^ received the same course of benzathine penicillin and immunotherapy with Bactek® (Inmunotek, Madrid, Spain). The route of administration was sublingual by means of spraying 2 puffs of 100 µl each (10^8^ bacteria/puff) daily, avoiding the concomitant intake of beverage or food. The delivered dose was maintained in the oral mucosa for a period of 2 minutes and then swallowed. The mean time of treatment was 4 (± 2) months per patient. Fifteen patients received treatment for a period of 3 months, 4 for a period of 6 months and 3 for 9 months. Clinical revisions in the medical records were in the first 4 months after the initiation of treatment and every 6 months until 3 years.

In both cases, it was considered a failure of the treatment (antibiotic or antibiotic plus Bactek®) the presence of 2 new episodes of tonsillitis after the initiation of the corresponding treatment.

All patients were warned, for report possible side effects such as itching, pain or edema oral and systemic

### Statistics

The Excel spreadsheet (Microsoft, Inc. USA) with the statistical add-in XLStat (Addinsoft, Paris, France) was used. Descriptive statistics of the age and the number of episodes of tonsillitis during the year before to the initiation of the treatment were expressed as the median with the interquartile (IQ) range). Mann-Whitney and Fisher’s exact tests were used for comparisons between the groups of patients that received Bactek® the group who did not.
